# PD-L1 Expression in Chinese Patients with Advanced Non-Small Cell Lung Cancer (NSCLC): A Multi-Center Retrospective Observational Study

**DOI:** 10.7150/jca.63003

**Published:** 2021-10-28

**Authors:** Xin Yang, Lili Jiang, Yan Jin, Peng Li, Yingyong Hou, Jingping Yun, Chunyan Wu, Wenyong Sun, Xiangshan Fan, Dong Kuang, Weiya Wang, Jinsong Ni, Anhua Mao, Wenmin Tang, Zhenhua Liu, Jiali Wang, Suijun Xiao, Yuan Li, Dongmei Lin

**Affiliations:** 1 Key Laboratory of Carcinogenesis and Translational Research (Ministry of Education), Department of Pathology, Peking University Cancer Hospital & Institute, Beijing, China; 2 Department of Pathology, West China Hospital, Sichuan University, Chengdu, Sichuan, China; 3 Department of Pathology, Fudan University Shanghai Cancer Center, Shanghai, China; 4 Department of Pathology, Sun Yat-sen University Cancer Center, Guangzhou, China; 5 Zhongshan Hospital of Fudan University, Shanghai, China; 6 Department of Pathology, Shanghai Pulmonary Hospital, Tongji University School of Medicine, Shanghai, China; 7 Zhejiang Cancer Hospital, Hangzhou, Zhejiang, China; 8 Nanjing Drum Tower Hospital, Nanjing, Jiangsu, China; 9 Department of Pathology, Tongji Hospital, Tongji Medical College, Huazhong University of Science and Technology, Wuhan, Hubei, China; 10 The first hospital of Jilin University - The Eastern Division, Changchun, Jilin, China; 11 Medical Affairs Department, MSD China, Shanghai, China

**Keywords:** non-small cell lung cancer, programmed death-ligand 1, immunohistochemistry, 22C3 antibody, driver mutations

## Abstract

**Objective:** This study aimed to investigate the prevalence of tumor programmed death-ligand 1 (PD-L1) expression in Chinese patients with advanced Non-Small Cell Lung Cancer (NSCLC).

**Methods:** Tumor tissues with histologically confirmed stage IIIB/IV NSCLC were retrospectively obtained from 10 centers in China. PD-L1 expression was determined using the PD-L1 IHC 22C3 pharmDx kit (Agilent, Santa Clara, CA, USA) and the samples were repetitively assayed with the PD-L1 IHC 22C3 Ab concentrate (Agilent, Santa Clara, CA, USA).

**Results:** Out of 901 patients who met the inclusion criteria, 879 (97.6%) had evaluable PD-L1 data. The number of patients with a PD-L1 tumor proportion score (TPS) < 1%, 1-49%, and ≥ 50% (corresponding to PD-L1 non-expression, low expression, and high expression) was 424 (48.2%), 266 (30.3%), and 189 (21.5%), respectively. PD-L1 expression was more likely to be found in patients younger than 75 years, men, current or former smokers, those with good performance status (PS) scores, and those with a wild-type epidermal growth factor receptor (EGFR). PD-L1 TPS ≥ 50% and ≥ 1% were respectively 28.0% and 50.2% among patients negative for both EGFR mutation and anaplastic lymphoma kinase (ALK) rearrangement. PD-L1 expression determined using the 22C3 antibody concentrate and pharmDx kit had comparable results.

**Conclusions:** The prevalence of PD‑L1 expression in advanced NSCLC was consistent with that reported in the global EXPRESS study. Age, gender, smoking history, PS scores, and EGFR/ALK mutation status affected PD-L1 expression. The 22C3 antibody concentrate appears to be an alternative reagent for the PD-L1 assay.

## Introduction

Lung cancer is the leading cause of cancer deaths globally 1, 2. However, the progress in precision therapy has contributed to a decline in related death rates. The annual mortality was reported to have declined by 5.9~6.3% in men and women with non-small cell lung cancer (NSCLC) since 2013 when biomarker-assisted targeted therapy was approved as a first-line regimen 1, 3. It is estimated that recent advances in immunotherapy will further boost the survival rates for lung cancer, especially for those without driver mutations 1. Indeed, immune checkpoint inhibitor (ICI)-based immunotherapy has become an important treatment modality for advanced NSCLC ever since it was approved by the Food and Drug Administration (FDA) in 2015. Single-agent immunotherapy or combination immunotherapy has dramatically improved long-term survival for advanced NSCLC 4-8.

Biomarkers for immunotherapy are helpful in decision making and identifying candidates who are more likely to respond to ICIs such as anti-programmed-cell death-1 (PD-1) or its ligand (PD-L1) monoclonal antibodies. PD-L1 expression is a rational biomarker for predicting the response to immunotherapy and the positive correlation between tumor PD-L1 expression and treatment response has been described in numerous clinical trials 9. The international KEYNOTE-001 study is the largest trial to validate the predictive value of tumor PD-L1 expression analyzed with an immunohistochemical (IHC) assay in lung cancer 10. The companion diagnostic PD-L1 IHC 22C3 pharmDx™ was subsequently approved to select NSCLC patients eligible for treatment with the PD-1 inhibitor pembrolizumab 11. For a panoramic understanding of the treatment candidates, the global EXPRESS study explored PD-L1 expression levels in NSCLC patients 12. Several other studies revealed that the prevalence of PD-L1 expression varied depending on different diagnostic patterns and populations. A recent meta-analysis including 50 studies concluded that PD-L1 expression was associated with several clinicopathological features and may serve as a poor prognostic biomarker for patients with lung cancer 13. However, limited data on PD-L1 expression are available for the Chinese NSCLC population, especially for the advanced stage.

The current study was conducted to obtain a profile of PD-L1 expression determined using 22C3 pharmDx™ in Chinese patients with advanced NSCLC. In addition, the performance of validated laboratory-developed tests (LDTs) using the 22C3 antibody concentrate with different platforms was evaluated in the study.

## Materials and methods

### Study design and objectives

This was a national, multicenter, retrospective, and observational study conducted in 10 centers in China aiming to evaluate the prevalence of PD-L1 protein on tumor tissue samples of advanced NSCLC.

The primary objective was to determine the prevalence of PD‑L1 expression in Chinese patients with stage IIIB/IV NSCLC using the PD‑L1 IHC 22C3 pharmDx™ kit. The secondary objectives were 1) to uncover the relationship between PD‑L1 expression and baseline demographic, clinicopathological, and genetic (epidermal growth factor receptor (EGFR) / anaplastic lymphoma kinase (ALK)) characteristics; 2) to assess the feasibility of PD-L1 expression measurement using the 22C3 antibody concentrate.

### Patient eligibility

The inclusion criteria were as follows: histologically confirmed primary stage IIIB/IV NSCLC; ≥ 18 years at diagnosis; treatment-naive tumor tissues available for PD-L1 measurement; and formalin-fixed paraffin-embedded (FFPE) tumor tissue block no more than 3 years old. All participants signed informed consent forms or had conditions that were accepted by the Institutional Review Board (IRB)/Ethical Review Committee (ERC) for waiving ICF.

### PD-L1 assessment

#### Sample

A total of five 4-5 μm FFPE sections from resected tissue, excisional biopsies, or core needle biopsies were required for each patient. Minimally, one matched hematoxylin and eosin (H&E) stained slide and two unstained slides for PD-L1 testing and the negative control were required. Samples were selected consecutively when possible.

### Measurement and platform

PD-L1 expression was determined using the PD-L1 IHC 22C3 pharmDx kit (Agilent, Santa Clara, CA, USA) on the Dako ASL48 platform. A total of 5x 4-5 μm freshly cut (within 1 month of assay, preferably within 1 week) FFPE sections from excisional biopsies or resected tissue or core needle biopsies are required for each patient. Two unstained slides (one for PD-L1 testing and one as a negative control) and one matched H&E stained slide are the minimum requirements; four unstained slides and one matched H&E stained slide are preferred to allow for rapid retest in the event that this is needed. PD-L1 IHC 22C3 pharmDx™ is a qualitative IHC assay using monoclonal mouse anti-PD-L1, clone 22C3. It is intended for use with the EnVision FLEX visualization system on the Dako ASL48 platform. PD-L1 protein expression is determined using a tumor proportion score (TPS), which is the percentage of viable tumor cells showing partial or complete membrane staining.

PD-L1 IHC 22C3 Ab concentrate (Agilent, Santa Clara, CA, USA) was additionally applied to some of the samples for testing on the Dako ASL48 platform or the Ventana BenchMark platform. More specifically, specimens were sectioned at a thickness of 3 μm and stained on positively charged glass slides stored at 4℃ within 3 days of sectioning. Deparaffinization, rehydration, and antigen retrieval were performed on PT Link (Dako PT100) using the EnVision™ FLEX Target Retrieval Solution at pH 6.0 for 53 minutes at room temperature. Following FLEX peroxidase block for 5 minutes, specimens were incubated with primary mouse anti-PD-L1 monoclonal antibody (ref. M365329; Dako, Inc.) using a concentration of 1:50 for 60 minutes at room temperature. Specimens were then incubated with the EnVision™ FLEX+ Mouse LINKER for 30 minutes at room temperature, followed by incubation with the EnVision™ FLEX HRP visualization reagent for 30 minutes at room temperature. Enzymatic conversion of the subsequently added 3,3'-diaminobenzidine tetrahydrochloride (DAB) chromogen for 10 minutes at room temperature followed by DAB enhancer for 5 minutes at room temperature resulted in the precipitation of a visible reaction product at the site of the antigen. The specimens were then counterstained with hematoxylin and coverslipped. All described steps were followed by buffer washes for 5 minutes (EnVisionTM FLEX Wash Buffer 20x). Each IHC run contained a positive control (on-slide tonsil tissue) and a negative antibody control (buffer, no primary antibody).

#### Quality control

Scoring was performed by certified pathologists at each site who attended organized pathology training programs on the detection of PD-L1 IHC before the study. The pathologists were blinded to the method (22C3 pharmaDx Kit or 22C3 antibody concentrate) used on individual slides and the order of the samples was determined by a technician. The samples were categorized as TPS ≥ 50% (high PD-L1 expression), 1-49% (low PD-L1 expression), ≥ 1% (PD-L1 expression), or < 1% (PD-L1 non-expression).

### Variables

Demographics, clinicopathological, and biomarker data were collected and recorded in a central database for analysis. Key variables included age at diagnosis, gender, Eastern Cooperative Oncology Group Performance Status (ECOG PS), smoking status, center, tumor stage, histology, site and type of tumor tissue samples, EGFR mutation status, and ALK translocation status. Information on the EGFR or ALK genetic status was retrospectively extracted from each center's electronic medical records system or by chart review. EGFR mutation was commonly measured using amplification refractory mutation system-polymerase chain reaction (ARMS-PCR), digital PCR, and next-generation sequencing (NGS). ALK rearrangement was screened with Ventana IHC, fluorescent *in situ* hybridization (FISH), reverse transcriptase (RT)-PCR, and NGS.

### Statistical analyses

Continuous data reported as the mean ± standard deviation or median (interquartile range) were compared with the t-test for comparisons between the two subgroups or one-way analysis for comparisons among the three subgroups. Categorical data expressed as a number with percentage (%) were compared with Pearson's Chi-Square test or Fisher's exact test if ≥ 25% of cells had expected counts < 5. A p-value < 0.05 was regarded as statistically significant.

## Results

### Patient screening and baseline characteristics

**Figure 1:**
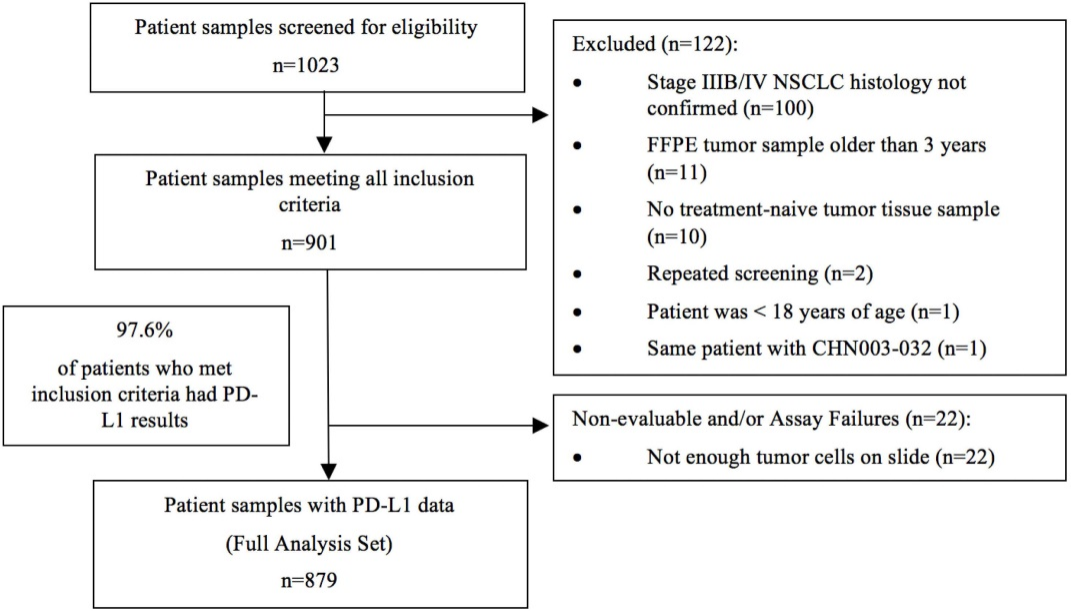
Study flow chart

The study started on January 1, 2019, with the first center initiation on August 15, 2019. Analysis of the last sample took place on June 29, 2020. A total of 1023 patients were initially screened. Out of 901 tumor samples that met the inclusion criteria, 879 samples (97.6%) had evaluable PD-L1 expression results and were included in the final analysis (Figure 1).

The median age of the overall population at diagnosis was 63.0 years (ranged: 30-90 years). The majority of the population were diagnosed at an age < 75 years (90.1%), were men (63.9%), had never smoked (50.9%), and had an ECOG PS score of 0-1 (70.2%). The tumor samples were mostly at stage IV (71.0%) and adenocarcinoma (71.7%). The percentage of samples from primary tumor tissues was 66.4% and 49.6% were from the core needle biopsy. Samples with ALK rearrangement accounted for 3.6% and those with EGFR mutation accounted for 23.9% in the entire population (Table 1). Two hundred and forty-eight samples were repetitively tested using the 22C3 antibody concentrate.

### Prevalence of PD-L1 expression

The number of samples that had PD-L1 non-expression, low expression, and high expression, defined as TPS < 1%, 1-49%, and ≥ 50%, was 424 (48.2%), 266 (30.3%), and 189 (21.5%), respectively.

The prevalence of high PD-L1 expression (TPS ≥ 50%) was higher in patients aged < 75 years (22.7%) compared with the prevalence in older patients (10.3%). Similarly, male patients were more likely to have high PD-L1 expression (25.6%) than female patients (14.2%). Current or former smokers tended to have a higher likelihood of exhibiting PD-L1 expression (TPS ≥ 1%: 58.5%) than never-smokers (47.0%). In addition, PD-L1 expression levels differed in patients with good and poor PS scores (p=0.0044). A marginally significant difference in PD-L1 expression was observed in samples with different histology results, with squamous cell carcinoma presenting a numerically higher proportion of PD-L1 expression than the non-squamous type (TPS ≥ 1%: 58.9% vs. 49.6%). The PD-L1 expression did not differ significantly in terms of TNM stage, primary or metastatic tissue, surgical or biopsy tissue, and different sampling locations (Table 2).

### Prevalence of PD-L1 in EGFR/ALK mutated NSCLC

No significant difference in PD-L1 expression prevalence was observed for patients with different ALK mutation states, wild-type, or ALK translocations (Table 3).

A slight difference was observed in EGFR mutant and wild-type groups when PD-L1 TPS ≥ 1% (43.8% versus 49.8%). However, EGFR mutation status seemed to affect the proportion of PD-L1 expression (p=0.0017). Patients with wild-type EGFR were more likely to have high PD-L1 expression than those with EGFR mutation (TPS ≥ 50%: 27.4% vs. 14.3%). Likewise, patients with wild-type ALK and EGFR status had a higher likelihood of showing PD-L1 expression than those with EGFR or ALK alterations (TPS ≥ 1%: 50.2% vs. 43.2%, TPS ≥ 50%: 28.0% vs. 13.2%) (Table 3).

In addition, the distribution of EGFR/ALK status in different PD-L1 expression categories showed that samples with wild-type EGFR or ALK mutation occupied a higher percentage than mutated samples in all PD-L1 expression categories (Table 4).

### PD-L1 expression using 22C3 antibody concentrate

Among 879 samples, 248 (28.2%) were eventually tested using the 22C3 antibody concentrate. Of the 248 samples evaluated, 99 (40.0%) were tested on the Dako ASL48 platform and 149 (60.1%) were tested on the Ventana BenchMark platform. When compared to the overall staining results obtained with the PD-L1 IHC 22C3 pharmDx^TM^ kit, LDT staining on the Dako ASL48 platform showed 100% concordance for TPS < 1%, a slightly lower rate for TPS of 1‑49% (23.2% for LDT and 24.2% for the overall staining method), and a slightly higher rate for TPS ≥ 50% (29.3% for LDT and 28.3% for the overall staining method). On the contrary, LDT staining on the Ventana BenchMark platform showed a higher rate for TPS < 1% (40.9% for LDT and 39.6% for the overall staining method) and TPS from 1‑49% (34.9% for LDT and 30.9% for the overall staining method) and a lower rate for TPS ≥ 50% (24.2% for LDT and 29.5% for the overall staining method).

## Discussion

The current real-world retrospective study explored the prevalence and features of PD-L1 tumor expression in a large-scale Chinese population with advanced NSCLC. We found that roughly half of the patients with advanced NSCLC (51.8%) had a PD-L1 expression TPS ≥ 1% and nearly one-fifth of patients (21.5%) had high PD-L1 expression (TPS ≥ 50%). PD-L1 expression was more likely to be found in individuals less than 75 years old, men, current/former smokers, those with good PS scores, and those with wild-type EGFR. PD-L1 expression determined using the 22C3 antibody concentrate and kit had comparable results.

The prevalence of positive PD-L1 expression in our Chinese cohort is largely consistent with that displayed in a global study. The EXPRESS study including tumor samples with a broad range of demographic and clinicopathological characteristics reported an overall 52% of PD-L1 TPS ≥ 1% and 22% of PD-L1 TPS ≥ 50% in patients with advanced NSCLC.^12^ The corresponding values in the current study were 51.8% of PD-L1 TPS ≥ 1% and 21.5% of PD-L1 TPS ≥ 50%.

Increasing evidence has demonstrated that PD-L1 expression related to aggressive behavior and disease progression may generally serve as a poor prognostic biomarker for patients with NSCLC 13. First, PD-L1 expression was observed more frequently in the advanced stage of NSCLC 14-16. For the NSCLC population in China, a previous small-scale study with surgically staged I-III samples showed that PD-L1 expression assessed using the PD-L1 IHC 22C3 antibody varied from 4.1% in adenocarcinomas to 34.3% in squamous cell carcinomas 17. In another study including 305 Chinese patients with stage I-IV NSCLC, the prevalence of PD-L1 expression assessed with the 22C3 assay was 46.6% with ≥ 1% as the cutoff and 20.7% with ≥ 50% as the cutoff 18. By contrast, the prevalence of PD-L1 expression, ≥ 1% or ≥ 50%, seems to be higher in the current study, partly due to the fact that all measured samples were from the advanced III/IV stage. Second, PD-L1 expression was more likely to present in the aggressive solid subtype of adenocarcinomas, which was correlated with worse survival 19, 20. It was also associated with poor tumor differentiation and positive lymph nodal metastasis 13. Third, in locally advanced stage NSCLC, PD-L1 expression predicted postoperative recurrence 21. Fourth, *in vivo* and *in vitro* experiments confirmed that PD-L1 functioned as a tumor-promoting factor in lung cancer 22. As single or combined immunotherapy has been approved for the first-line treatment of advanced NSCLC, our findings represent the real percentage of candidates who might benefit from indications for immunotherapy 4, 6, 7, 23, 24.

There are controversies regarding the relationship between PD-L1 expression and demographic and clinicopathological features, which may be attributed to the population heterogeneity in various studies. Some earlier studies did not support an association between PD-L1 expression and gender, age, smoking history, or PS score 25. We found that positive PD-L1 expression was more frequently observed in men, current/former smokers, and individuals with good PS scores. These findings were inconsistent with some previous reports 18, 26. Tobacco exposure induces chronic inflammation and upregulates interferon-γ, which triggers increased PD-L1 expression 27, 28. More specifically, higher daily cigarette consumption, longer smoking duration, and current smoking status were significantly associated with high PD-L1 expression 18. Considering that men tend to smoke at higher rates than women, it is not surprising that men had a greater percentage of positive PD-L1 expression, as confirmed in this study 26, 29.

EGFR- or ALK-tyrosine kinase inhibitor (TKI) treatment is an important targeted therapy for lung adenocarcinoma in East Asian patients because the overall EGFR mutation frequency is 47.9% in Asian patients and only 19.2% in Western patients 30, 31. However, there is insufficient evidence to support the application of mono-immunotherapy for NSCLC patients who fail to respond to frontline TKI therapy 32-34. The correlations between the EGFR/ALK mutation and PD-L1 expression may provide some clues for immunotherapy in this subgroup. Our findings validated previous findings that NSCLC patients with EGFR mutation had a lower chance of PD-L1 expression than those with wild-type EGFR 26, 33-36. In the current study, 62 (29.5%) NSCLC patients with EGFR mutations had a PD-L1 expression of 1%-49% and 30 (14.3%) had PD-L1 expression ≥ 50%. The proportion of positive PD-L1 expression was similar to previous findings 37. The overlap between PD-L1 expression and EGFR mutations is still considerable based on the results from a previous study in Chinese lung adenocarcinoma patients 19. The subgroup of lung adenocarcinomas patients with co-occurrence of aberrations in classical therapeutic genes such as EGFR mutation and PD-L1 positive expression accounted for 9.6% of Chinese adenocarcinoma patients 19. Therefore, whether immunotherapy combined with chemotherapy or anti-angiogenesis agent could be an effective option for EGFR-mutated NSCLC after exhaustion of targeted therapies is under research in ongoing KEYNOTE-789 (NCT03515837) and Checkmate-722 (NCT02864251) and ORIENT-31 (NCT03802240) studies. Another issue worth clarifying is that the EGFR/ALK mutation status was retrospectively collected in this study. Among the available detection methods, NGS may have advantages over PCR by providing a higher mutation detection rate and a wider spectrum. However, given that more than 90% of detected EGFR mutations were common mutations of 19del or L858R that could be well identified by all methods; we speculate that our findings are applicable in different settings 38.

Although a lower PD-L1 positive rate was associated with EGFR mutation, NSCLC patients with ALK rearrangement were reported to possess higher PD-L1 expression 36, 39. Research findings showing that the EML4-ALK oncoprotein can upregulate PD-L1 expressions in lung cancer cells explain this result 40, 41. Recent studies showed that 50%-55.6% of ALK-positive NSCLC patients were PD-L1-positive (TPS ≥ 1%) and 16-30.6% had high expression (TPS ≥ 50%) 39, 42. We also observed a high percentage of TPS ≥ 50% (21.9%) in ALK-rearranged NSCLC. However, no significant difference was seen between patients with wild-type or rearranged ALK status. In addition, clinical trials demonstrated that the combination of ALK-TKI and immunotherapy may lead to higher toxicity 43. Whether a higher rate of positive PD-L1 expression implies a higher possibility of immunotherapy remains unclear for ALK-positive NSCLC patients.

The occurrence of brain metastasis in NSCLC is steadily increasing with advances in therapeutic strategies 44, 45. The prognosis of patients with brain metastases remains poor 46. Patients with brain metastases are mostly excluded from pivotal clinical trials and the prevalence of PD-L1 expression in metastatic brain tissue is largely unknown. In this study, we obtained 58 brain metastatic tissue samples, 44.8% of which exhibited PD-L1 expression (TPS ≥ 1%) while 20.7% exhibited high expression (TPS ≥ 50%). The expression pattern of PD-L1 did not differ from those of other sample tissues. A recent study reported concordant PD-L1 expression between brain metastases and primary tumors 47. However, the prognostic role of PD-L1 expression in brain metastases has not been fully discovered although encouraging results suggest that immunotherapy may be active in the central nervous system (CNS) in NSCLC patients with high PD-L1 expression 48, 49.

PD-L1 IHC 22C3 pharmDx is a companion diagnostic test for PD-L1 detection. Different PD-L1 assays and evaluation systems for PD-L1 expression may produce discrepant results 50, 51. Other IHC assays using antibodies instead of the 22C3 clone, for example, 28-8, SP263, and SP142, have been developed to evaluate PD-L1 expression and are commercially available. High concordance for PD-L1 staining can be achieved between PD-L1 IHC 22C3 pharmDx assay and LDTs 52. Additionally, both Ventana stains (UltraView and OptiView) have a high correlation with the PD-L1 IHC 22C3 pharmDx kit 53. Part of the tumor samples in this study were tested using 22C3 antibody concentrate. The findings that PD-L1 expression determined using the 22C3 antibody concentrate on the Dako ASL48 platform and Ventana BenchMark Platform was comparable to that determined with validated LDTs support the feasibility of the clinical application of these methods.

There are several limitations in this study. First, the retrospective nature limited the comprehensiveness of the data. Second, we did not further evaluate the association between different subtypes of EGFR/ALK mutations and PD-L1, and other important driver genes such as KRAS were not analyzed. Third, the concordance of the 22C3 antibody concentrate and traditional assay kit on different platforms is worthy of further validation in larger sample sets.

In conclusion, the prevalence of PD‑L1 expression in Chinese patients with advanced NSCLC is consistent with that reported in the global EXPRESS study. Age, gender, smoking history, PS scores, and EGFR/ALK mutation status affected PD-L1 expression. The 22C3 antibody concentrate may be an alternative regent for the 22C3 pharmDx assay.

## Author contribution

Yan Jin, Jingping Yun, Chunyan Wu, Wenyong Sun, Anhua Mao, Wenmin Tang, Suijun Xiao, Yuan Li and Dongmei Lin conceived, designed, or planned the study. Xin Yang, Lili Jiang, Yan Jin, Peng Li, Yingyong Hou, Jingping Yun, Chunyan Wu, Wenyong Sun, Xiangshan Fan, Dong Kuang, Weiya Wang, Jinsong Ni and Yuan Li collected and assembled the data. Yan Jin, Jingping Yun, Weiya Wang, Zhenhua Liu, Jiali Wang, Suijun Xiao, Jinsong Ni, Yuan Li and Dongmei Lin performed or supervised the analyses. Lili Jiang, Jingping Yun, Xiangshan Fan, Weiya Wang, Jinsong Ni, Zhenhua Liu, Jiali Wang, Suijun Xiao, Yuan Li and Dongmei Lin interpreted the results. Yan Jin, Jingping Yun and Yuan Li wrote sections of the initial draft. Anhua Mao and Yuan Li provided administrative, technical, and logistic support. All authors provided substantive suggestions for revisions, reviewed and approved the final version of the paper, and ensured the accuracy of all aspects of the work.

## Figures and Tables

**Table 1:** Demographic and clinicopathological characteristics of the study population (N=879)

Category	N (%)
Age at diagnosis	
Median	63.0
Min, Max	30, 90
Age at diagnosis	
< 75	792 (90.1)
≥ 75	87 (9.9)
Age at diagnosis	
< Median	402 (45.7)
≥ Median	477 (54.3)
Gender	
Male	562 (63.9)
Female	317 (36.1)
Race	
Chinese	878 (99.9)
Other	1 (0.1)
Smoking status	
Never	447 (55.8)
Former	209 (26.1)
Current	145 (18.1)
ECOG Performance Status	
0-1	617 (92.6)
≥ 2	49 (7.4)
AJCC Stage	
IIIB	225 (25.6)
IV	624 (71.0)
Others	30 (3.4)
Histology subtype	
Squamous cell carcinoma	207 (23.5)
Adenocarcinoma	630 (71.7)
Others	42 (4.8)
Site	
Primary tumor	584 (66.4)
Metastatic lesion	295 (33.6)
Sampling location of metastatic lesion	
Lymph node	134 (15.2)
Brain	58 (6.6)
Liver	9 (1.0)
Others	94 (10.7)
Tissue type	
Surgical resection	314 (35.7)
Biopsy (Core needle biopsy + Bronchial Biopsy + Pleura Biopsy + EBUS + TBNA)	511 (58.1)
Others	54 (6.1)
ALK status	
Rearrangement	32 (6.0)
Wild type	500 (94.0)
EGFR mutation status	
Mutant	210 (43.1)
Wild type	277 (56.9)
Joint ALK/EGFR Status	
ALK+ or EGFR+	190 (45.8)
ALK-/EGFR-	225 (54.2)

ECOG: Eastern Cooperative Oncology Group; AJCC: American Joint Committee on Cancer; EGFR: epidermal growth factor receptor; ALK: anaplastic lymphoma kinase; EBUS-TBNA: endobronchial ultrasound-guided transbronchial fine needle aspiration

**Table 2:** Prevalence of PD-L1 TPS by baseline demographic and clinicopathologic characteristics

Category, N (%)	TPS < 1%	TPS 1-49%	TPS ≥ 50%	P-value
Total	424 (48.2)	266 (30.3)	189 (21.5)	
Age at diagnosis				0.0149
< 75	379 (47.9)	233 (29.4)	180 (22.7)	
≥ 75	45 (51.7)	33 (37.9)	9 (10.3)	
Gender				0.0002
Male	250 (44.5)	168 (29.9)	144 (25.6)	
Female	174 (54.9)	98 (30.9)	45 (14.2)	
Smoking status				< 0.0001
Never	237 (53.0)	140 (31.3)	70 (15.7)	
Current/Former	147 (41.5)	110 (31.1)	97 (27.4)	
ECOG Performance Status				0.0044
0-1	317 (51.4)	152 (24.6)	148 (24.0)	
≥ 2	24 (49.0)	21 (42.9)	4 (8.2)	
AJCC Stage				0.0927
IIIB	95 (42.2)	73 (32.4)	57 (25.3)	
IV	314 (50.3)	184 (29.5)	126 (20.2)	
Histology subtype				0.0635
Squamous cell carcinoma	85 (41.1)	71 (34.3)	51 (24.6)	
Non- Squamous	331 (50.4)	193 (29.4)	133 (20.2)	
Site				0.1185
Primary tumor	273 (46.7)	190 (32.5)	121 (20.7)	
Metastatic lesion	151 (51.2)	76 (25.8)	68 (23.1)	
Tissue type				0.1899
Surgical resection	161 (51.3)	82 (26.1)	71 (22.6)	
^*^Biopsy	241 (47.2)	164 (32.1)	106 (20.7)	
Sampling location				0.3803
Lymph node	67 (50.0)	32 (23.9)	35 (26.1)	
Brain	32 (55.2)	14 (24.1)	12 (20.7)	
Liver	6 (66.7)	0	3 (33.3)	
^#^Others	46 (48.9)	30 (31.9)	18 (19.2)	

TPS: tumor proportion score; ECOG: Eastern Cooperative Oncology Group. * including biopsy sample from core needle biopsy, bronchial Biopsy, pleura biopsy, or EBUS/TBNA. # Others indicate samples from the adrenal gland, gallbladder, gastrointestinal tract, heart, kidney, lung, breast, skin, pancreas, spleen, thyroid, pleura, soft tissue, and other organs.

**Table 3:** Prevalence of PD-L1 in EGFR/ALK-mutated NSCLC

Category, N (%)	TPS < 1%	TPS 1-49%	TPS ≥ 50%	P-value
ALK status				0.9947
Rearrangement	17 (53.1)	8 (25.0)	7 (21.9)	
Wild type	261 (52.2)	128 (25.6)	111 (22.2)	
EGFR mutation status				0.0017
Mutant	118 (56.2)	62 (29.5)	30 (14.3)	
Wild type	139 (50.2)	62 (22.4)	76 (27.4)	
Joint ALK/EGFR Status				0.0009
ALK^+^ or EGFR^+^	108 (56.8)	57 (30.0)	25 (13.2)	
ALK^-^ and EGFR^-^	112 (49.8)	50 (22.2)	63 (28.0)	

TPS: tumor proportion score; EGFR: epidermal growth factor receptor; ALK: anaplastic lymphoma kinase

**Table 4:** Distribution of EGFR/ALK status in different PD-L1 expression categories

Category, N (%)	Total	EGFR			ALK		
		mutant	wild type	no data	rearrangement	wild type	no data
TPS < 1%	424	118 (27.8)	139 (32.8)	167 (39.4)	17 (4.0)	261 (61.6)	146 (34.4)
TPS ≥ 1%	455	92 (20.2)	138 (30.3)	225 (49.5)	15 (3.3)	239 (52.5)	201 (44.2)
TPS ≥ 50%	189	30 (15.9)	76 (40.2)	83 (43.9)	7 (3.7)	111 (58.7)	71 (37.6)

## Abbreviations

NSCLC: non-small cell lung cancer
ICI: immune checkpoint inhibitor
FDA: Food and Drug Administration
PD-1: programmed-cell death-1
PD-L1: programmed-cell death-1 ligand
IHC: immunohistochemical
LDTs: validated laboratory-developed tests
EGFR: epidermal growth factor receptor
ALK: anaplastic lymphoma kinase
FFPE: formalin-fixed paraffin-embedded
TPS: tumor proportion score
ECOG PS: Eastern Cooperative Oncology Group Performance Status
CNS: central nervous system
